# Yeast microcapsules encapsulating metal-phenolic nanozymes alleviate ulcerative colitis by mitigating oxidative stress and modulating the gut microbiota

**DOI:** 10.1016/j.mtbio.2025.101902

**Published:** 2025-05-23

**Authors:** Meihong Chai, Yuanyuan Zhu, Liyuan Chen, Shanli Zhang, Yahui Huang, Mingzhen Zhang, Weiwei Jin

**Affiliations:** a Department of Pharmacy, Xi'an Hospital of Traditional Chinese Medicine, Xi'an, Shaanxi, 710021, China; b School of Basic Medical Sciences, Xi'an Jiaotong University, Xi'an, Shaanxi, 710061, China; c General Surgery, Cancer Center, Department of Gastrointestinal and Pancreatic Surgery, Zhejiang Provincial People's Hospital (Affiliated People's Hospital), Hangzhou Medical College, Hangzhou, Zhejiang, 310014, China

**Keywords:** Ulcerative colitis, Metal-phenolic nanozyme, Yeast microcapsules, Oxidative stress, Gut microbiota

## Abstract

Ulcerative colitis (UC) is defined as a chronic intestinal inflammation with an unknown cause. During its occurrence and development, oxidative stress and intestinal microbiota dysbiosis play important roles. Nevertheless, the treatment of UC continues to pose significant challenges due to the intricate nature of physiological barriers and the suboptimal targeting efficacy of traditional therapeutic strategies. To solve the dilemma facing UC treatment, in this study, we developed a metal-phenolic nanozyme, designated as DHM-Zn, which exhibits anti-inflammatory and antioxidant properties via metal coordination between dihydromyricetin (DHM) and Zn^2+^. Furthermore, we engineered yeast microcapsules (YM) encapsulating the metal-phenolic nanozymes (DZ@YM), leveraging the inherent biosafety and tolerability advantages offered by natural microorganisms. Following oral administration, the intestinal retention characteristics of YM facilitated the efficient aggregation of DHM-Zn nanozymes at the inflammation site, thereby extending their therapeutic efficacy. In addition to augmenting anti-inflammatory and antioxidant effects, DZ@YM contributed to the restoration of intestinal microbial balance by increasing the abundance of beneficial bacteria such as *Parabacteroides* and *Muribaculaceae*, while regulating potentially harmful bacteria like *Clostridium-sensu-stricto* and *Escherichia-Shigella*, thereby achieving a synergistic multi-pathway therapeutic approach. Collectively, with excellent biocompatibility, this novel therapeutic approach demonstrates extensive potential for clinical application in the treatment of UC and offers new directions and insights for UC therapy.

## Highlights

• DHM-Zn demonstrates enzymatic activities and can scavenge multiple free radicals.
• DZ@YM colonizes the inflamed colon, restoring redox balance and reducing inflammation.
• DZ@YM alleviates colitis by restoration of intestinal microbial balance.

## Graphical abstract

**Figure:**
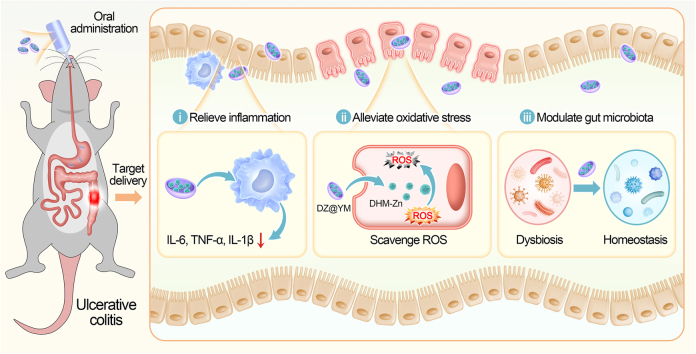

## 1 Introduction

Ulcerative colitis (UC) is a chronic and nonspecific inflammatory disorder of the intestine, the underlying etiology of which remains incompletely understood. Clinically, it is characterized by persistent or relapsing episodes of diarrhea accompanied by mucopurulent bloody stools and associated abdominal pain [1]. With the continuous advancement of society, the incidence rate of UC has been showing an upward trend globally. Notably, in Western countries, there has been a particularly significant increase in the prevalence of this disease [2]. Additionally, newly industrialized regions such as Asia, South America, and the Middle East have also witnessed a rising incidence, making UC a global health concern [1]. The exact cause of UC remains unclear; however, the most widely accepted theory suggests that genetic factors, immune dysregulation, changes in the gut microbiome, and environmental triggers together disrupt intestinal homeostasis, resulting in chronic inflammation [[3], [4], [5], [6]]. Clinically, therapeutic strategies vary based on the stage and severity of UC, aiming to alleviate symptoms. While active medical therapy can provide symptomatic relief, persistent therapeutic limitations exist regarding long-term efficacy, including high recurrence rates, significant adverse effects, and increasing drug resistance. In cases of severe or refractory disease, surgical intervention is often contemplated; however, this approach carries substantial operative risks and may potentially compromise patients’ quality of life [7]. Consequently, there exists an urgent need for novel therapeutic strategies that demonstrate enhanced safety profiles and improved clinical effectiveness in managing UC.

Dihydromyricetin (DHM), a flavonoid compound derived from the plant *Ampelopsis grossedentata*, exhibits potent anti-inflammatory and antioxidant properties [[8], [9], [10]]. However, its clinical application is limited by low solubility, poor permeability, gastrointestinal instability, and low bioavailability [11]. The unique chemical architecture of polyphenolic compounds facilitates the formation of metal-phenolic coordination complexes, thereby improving their aqueous solubility [12]. Importantly, these metal-phenolic coordination complexes exhibit enzyme-mimetic catalytic properties that offer multiple advantages over conventional natural enzymes, including cost-effectiveness, enhanced stability, chemical tolerability, and adjustable catalytic performance, while maintaining structural modularity for functional modifications [13,14]. Of particular therapeutic significance, redox-active nanozymes with intrinsic antioxidant capacity demonstrate efficacy in concurrently mitigating oxidative stress and attenuating pro-inflammatory signaling pathways, thereby positioning these nanomaterials as promising therapeutic candidates for UC management.

The colon exhibits evolutionarily conserved adaptations. Due to its gradient-dependent hypoxic microenvironment and the dynamics regulated by peristalsis, it facilitates the colonization of microorganisms [15]. Alterations in gut microbiota, a critical component of the intestinal microecosystem, can lead to significant physiological changes and contribute to disease states [16]. In UC patients, gut microbiota exhibits reduced diversity, increased harmful bacteria, and decreased beneficial bacteria, leading to impaired intestinal mucosal barrier function and subsequent inflammation [17,18]. Moreover, changes in gut microbiota can disrupt the balance of the intestinal immune system, resulting in abnormal immune regulation and exacerbating inflammation. Therefore, restoring the balance of gut microbiota has emerged as a crucial approach for treating UC [[19], [20], [21]].

Yeast microcapsules (YM), acting as a natural oral microcapsule carrier, exhibit excellent biocompatibility, primarily composed of β-glucan, ensuring stability within the complex gastrointestinal environment and resistance to gastric acid and digestive enzymes [22,23]. The hollow interior and porous surface facilitate drug loading and enhance drug stability. Notably, YM contains mannan, which possesses prebiotic functionality, promoting the proliferation of beneficial bacteria while inhibiting harmful bacteria, thus demonstrating significant therapeutic potential in regulating gut microbiota [[24], [25], [26]].

**Fig. 1:**
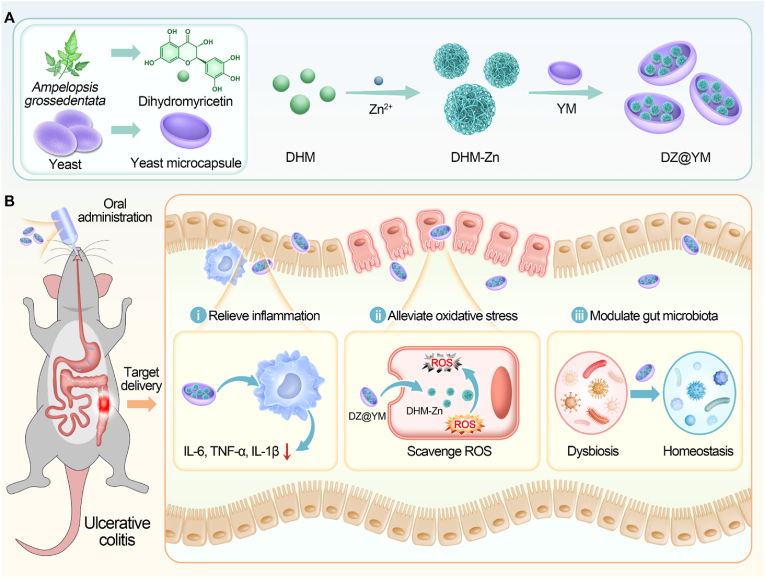
**The synthesis of DZ@YM and its mechanism in the treatment of UC.** (A) Preparation of DZ@YM. (B) The intestinal targeting ability of YM increased the concentration and duration of DZ@YM at the site of inflammation, and DZ@YM improved ulcerative colitis by relieving inflammation, alleviating oxidative stress, and modulating gut microbiota.

In this study, zinc ions were successfully complexed with dihydromyricetin to prepare DHM-Zn nanozymes, which possess both anti-inflammatory and antioxidant properties. Both *in vitro* and *in vivo* investigations have validated that DHM-Zn nanozymes effectively alleviate oxidative stress at inflammatory loci through reactive oxygen species (ROS) scavenging. Furthermore, these nanozymes demonstrate immunomodulatory capabilities by modulating pro-inflammatory cytokine expression profiles and impeding M1 macrophage polarization, thereby attenuating inflammatory cascades. To enhance the localized accumulation and prolong the therapeutic duration of DHM-Zn nanozymes at pathological sites, we engineered YM-encapsulated DHM-Zn nanozymes (DZ@YM). The unique intestinal retention property of YM allows DZ@YM to enhance its anti-inflammatory and antioxidant effects. Additionally, DZ@YM optimizes gut microbiota structure by increasing the abundance of beneficial bacteria such as *Parabacteroides* and *Muribaculaceae*, while regulating potentially harmful bacteria like *Clostridium-sensu-stricto* and *Escherichia-Shigella*, achieving a balanced gut microbiota composition and facilitating multi-targeted combined treatment effects (Fig. 1). This novel therapeutic approach demonstrates extensive potential for clinical application in the treatment of UC and offers new directions and insights for UC therapy.

## 2 Materials and methods

### 2.1 Materials

Dihydromyricetin, (CH_3_COO)_2_Zn•2H_2_O, DPPH, TMB, and MTT were purchased from Macklin (Shanghai, China). Active dry yeast was purchased from Angel Yeast (Yichang, China). LPS (from *Escherichia coli* O55: B5) and reactive oxygen species assay kit were purchased from Solarbio (Beijing, China). The fluorescent dye DiR and DHE were purchased from UElandy (Suzhou, China). ABTS was purchased from Beyotime Biotechnology (Shanghai, China). SOD assay kit-WST was purchased from Dojindo (Shanghai, China). DSS was purchased from MP Biomedicals (Santa Ana, CA, USA). PE anti-mouse CD86 antibody (105007) and APC anti-mouse CD206 antibody (141708) were purchased from BioLegend (San Diego, CA).

### 2.2 Preparation of DHM-Zn

64 mg of DHM was dissolved in 100 mL of absolute ethanol. Subsequently, the pH of the solution was adjusted to 7.5 by using sodium acetate. After the pH adjustment, the solution was stirred for 30 min. Then, 100 mL of a 2 mM zinc acetate ethanol solution was slowly added. The resulting mixture was stirred and subjected to reflux at a constant temperature of 70 °C for 6 h. Following the reflux process, the precipitate was separated by centrifugation at 5000 rpm for 10 min. After centrifugation, the obtained solution was thoroughly washed with absolute ethanol several times. Finally, the resulting precipitate was DHM-Zn.

### 2.3 Preparation of DZ@YM

20 g of yeast was suspended in 300 mL of 1 M NaOH solution, undergoing thermal processing at 80 °C for 60 min under reflux conditions. Subsequently, the yeast was resuspended in a buffered acidic aqueous medium (pH = 4) and maintained at 60 °C for 60 min. The precipitate was then collected by centrifugation at 3000 rpm for 10 min. The precipitate was collected by centrifugation after rinsing the precipitate with isopropanol several times and again with acetone. The resulting precipitate was YM.

1 mL of DHM-Zn with a concentration of 10 mg/mL was added to the YM of the same concentration. The mixture was then subjected to sonication for 30 min, followed by stirring for 2 h. Subsequently, it was centrifuged at 3000 rpm for 10 min to obtain a precipitate. The precipitate was dispersed with ultrapure water and centrifuged again to remove the unencapsulated DHM-Zn. The resulting product was DZ@YM.

### 2.4 Characterization of DHM-Zn and DZ@YM

The morphologies of DHM-Zn, YM, and DZ@YM were characterized by transmission electron microscopy (TEM, JEOL JEM-2100). The ultraviolet–visible absorption spectra (UV–Vis) and Fourier transform infrared spectra (FTIR) of DHM and DHM-Zn were recorded by an ultraviolet spectrophotometer (Shimadzu UV2700) and a Fourier transform infrared spectrophotometer (Thermo-Scientific Nicolet 5700). The hydrodynamic diameters and zeta potentials of DHM-Zn and DZ@YM were monitored by dynamic light scattering (DLS, Malvern Zetasizer Nano ZS90). The elemental contents and valence states of DHM-Zn were characterized by an X-ray photoelectron spectrometer (XPS, Thermo Fisher ESCALAB Xi+).

### 2.5 ROS scavenging evaluation of DHM-Zn

ABTS experiment: A 10 μL aliquot of serially diluted DHM-Zn solutions was mixed with 190 μL of ABTS working solution to achieve final DHM-Zn concentrations of 5, 10, 25, 50, and 100 μg/mL. Following a 5-min room temperature incubation period, absorbance measurements were conducted at 734 nm using spectrophotometric detection.

DPPH experiment: DHM-Zn solutions were serially diluted, and equal-volume aliquots were combined with 125 μM DPPH ethanolic solution, resulting in final DHM-Zn concentrations of 5, 10, 25, 50, and 100 μg/mL. After allowing the reaction to proceed for 30 min under ambient conditions, radical scavenging activity was quantified spectrophotometrically through absorbance measurements at 517 nm.

TMB experiment: Equal volumes of a 10 μM FeCl_2_ solution, a 50 μM H_2_O_2_ solution, and a 300 μM TMB solution were mixed, and 10 μL of a DHM-Zn solution with different concentrations was mixed with 90 μL of the mixed solution. The final concentrations of DHM-Zn were 5, 10, 25, 50, and 100 μg/mL, respectively. After a 30-min reaction period at ambient temperature, the absorbances at 645 nm were measured.

NBT experiment: A mixture containing 50 μM NBT, 13 mM L-methionine, and 20 μM riboflavin was prepared with 25 mM PBS buffer. Then, 10 μL of DHM-Zn solution was added to 90 μL of the above solution, and the final concentrations of DHM-Zn were 5, 10, 25, 50, and 100 μg/mL, respectively. After 5 min of LED light irradiation, the absorbances at 560 nm were measured.

ESR experiment: BMPO exhibits specific spin-trapping efficacy for O_2_^•-^ generated during the enzymatic conversion of hypoxanthine by xanthine oxidase. Similarly, DMPO has been shown to capture •OH, while DPPH radical and •OH production are the methods mentioned above in the DPPH experiment and the TMB experiment. The resulting radicals were then utilized to record signal changes using ESR (EPR 200 M, CIQTEK Co., Ltd.).

Stability experiment: 1 mg of DHM-Zn was weighed and thoroughly mixed with 1 mL of commercially available simulated intestinal fluid (SIF, Beijing Leagene Biotechnology Co., Ltd.), and then incubated at a constant temperature of 37 °C for 2 h. For the simulated inflammatory colonic fluid (SICF) system, H_2_O_2_ with a concentration of 200 μM was added to 1 mL of commercially available simulated colonic fluid (COOLABERSCIENCE &TECHNOLOGY Co., Ltd.) to construct an inflammatory environment. After the incubation was completed, the free radical scavenging ability of DHM-Zn in different simulated body fluid environments was determined by using the above-mentioned free radical scavenging experimental method.

### 2.6 Cell culture

The medium used for RAW 264.7 cells is the RAW 264.7 cell-specific medium, which consists of DMEM medium supplemented with 10 % fetal bovine serum (FBS) and 1 % penicillin/streptomycin. The medium for FHC cells is DMEM medium enriched with 1 % penicillin/streptomycin and 10 % FBS. The culture conditions are maintained in an environment of 37 °C and 5 % CO_2_.

### 2.7 Intracellular anti-inflammatory and antioxidant effects

Following the co-culturing of RAW 264.7 cells with 40 μg/mL DHM or DHM-Zn for a period of 8 h, the medium was subjected to the addition of 600 μM H_2_O_2_ for a further 1 h of co-culturing. Thereafter, the medium was discarded and the cells were co-cultured with medium containing 10 μM DCFH-DA or 1 μM DHE for a further 1 h. Subsequently, the cells were washed three times with PBS to remove residual probes, intracellular ROS levels were assessed through fluorescence microscopic imaging and flow cytometric quantification.

RAW 264.7 cells were separately co-incubated with 40 μg/mL of DHM or DHM-Zn for a duration of 8 h. After the incubation was completed, lipopolysaccharide (LPS) with a final concentration of 1 μg/mL was added to the cell system, and then the co-incubation process was continued for 12 h. Cellular samples were collected for RNA isolation, followed by cDNA synthesis and quantitative real-time PCR (qPCR) analysis of pro-inflammatory mediator expression. Parallel samples underwent CD86 or CD206 immunostaining (200 μL, 4 °C, 30 min) with PBS washing three times preceding flow cytometric quantification.

### 2.8 Animals

Female C57BL/6 mice were provided by the Medical Experimental Animal Center of Xi'an Jiaotong University, Shaanxi Province, China. All experiments complied with the Institutional Animal Care and Use Committee at Xi'an Jiaotong University.

### 2.9 The intestinal targeting and retention effects of DZ@YM

The preparation of DiR-labeled DHM-Zn involved the overnight stirring of 20 μL of 10 mM DiR with 5 mg/mL DHM-Zn. Subsequently, DiR-labeled DHM-Zn was thoroughly stirred with YM overnight, and the DiR-labeled DZ@YM was obtained by centrifugation at 3000 rpm for 10 min. Following oral gavage administration of DiR-labeled DHM-Zn and DZ@YM, in vivo fluorescence imaging was performed at predetermined intervals. At the 24-h endpoint, animals were euthanized for ex vivo quantification of tissue-specific fluorescence signals. In parallel experimental cohorts, UC was induced through 2.5 % DSS oral administration, with subsequent comparative biodistribution analysis conducted using identical fluorescence tracking methodology.

### 2.10 Biosafety assessment

In a 96-well cell culture plate, RAW 264.7 cells or FHC cells were seeded in each well (10,000 cells/well). Then, different concentrations of DHM-Zn were added, and the co-incubation was carried out under standard cell culture conditions for 24–48 h. After the incubation, the original culture medium in each well was gently discarded, and fresh medium containing 5 mg/mL MTT was added to each well. The incubation was continued in the dark for 4 h. After that, the MTT medium was carefully removed, and 100 μL of dimethyl sulfoxide (DMSO) was added to each well. The plate was shaken on a horizontal shaker for 10 min. Finally, the absorbance values of each well were measured at a wavelength of 490 nm to quantify the cell viability level. In addition, to systematically evaluate the biosafety of YM and DZ@YM, the same experimental protocol was adopted. RAW 264.7 cells were co-incubated with different concentrations of YM and DZ@YM, respectively, for 24–48 h, and the changes in cell viability were detected by the MTT method to assess their potential cytotoxicity.

To explore the short-term and long-term toxic effects of DHM-Zn and DZ@YM, mice were administered DHM-Zn and DZ@YM via daily oral gavage at a dose of 40 mg/kg. The short-term toxicity experiment lasted for 7 days, while the long-term toxicity experiment extended over 30 days. Mice were sacrificed on the day following the completion of gavage. Blood samples were collected and aliquoted for complete blood cell count analysis and serum biochemical index detection, aiming to assess the impacts on the blood system and major organ functions. Simultaneously, primary tissues including the heart, liver, spleen, lung, and kidney were collected, fixed in 4 % paraformaldehyde (PFA) solution, and then comprehensively evaluated for pathological morphological changes using hematoxylin-eosin (H&E) staining.

### 2.11 Colitis model

C57BL/6 mice aged between six and eight weeks were randomized into six experimental cohorts (n = 8/group) for DSS-induced ulcerative colitis modeling. Following a week of acclimation, the mice were administered 2.5 % DSS to induce acute ulcerative colitis. Thereafter, over seven days, the mice were given 40 mg/kg of DHM, DHM-Zn, YM, and DZ@YM by oral gavage for five consecutive days. The body weight, stool condition, and hematochezia of the mice were recorded daily. The mice were sacrificed on the final day, and their spleens and colons were removed for subsequent experimental analysis.

### 2.12 Analysis of gut contents microbiota

In the gut contents microbiota analysis experiment, gut content samples were collected and immediately stored at −80 °C to preserve the integrity of the microbial community structure. The bacterial 16S rDNA sequencing was entrusted to LC-Bio Technologies Co., Ltd. The detailed experimental procedures were as follows: First, microbial DNA was efficiently extracted from fecal samples. Subsequently, the V3-V4 hypervariable regions of 16S rDNA were specifically amplified using the universal primers 341F/805R. The purified PCR products were subjected to high-throughput sequencing on the Illumina NovaSeq 6000 platform (in PE250 mode) to obtain raw sequence data. For the raw data, pre - processing steps including adapter sequence removal, quality filtering, and chimera elimination were successively performed to ensure data quality. The DADA2 software was utilized for in-depth analysis to generate amplicon sequence variants (ASVs), which were then compared with the SILVA/NT-16S authoritative database for taxonomic annotation. The QIIME2 software was employed to conduct α-diversity and β-diversity analyses, comprehensively evaluating the richness, evenness of the microbiota and the structural differences in microbiota among samples. The Wilcoxon rank-sum test and linear discriminant analysis effect size (LEfSe) were used to systematically identify bacterial genera with significantly different abundances among groups. The analysis results were visualized using the R language plotting packages.

### 2.13 Statistical analysis

All experimental datasets underwent parametric analysis using GraphPad Prism 9.0. Two-tailed unpaired Student's t-test was applied for intergroup comparisons between two experimental cohorts, whereas one-way analysis of variance (ANOVA) was executed for multi-group comparisons. The results were expressed as mean ± standard deviation (SD), and the significant differences between groups were expressed as ∗*p* < 0.05, ∗∗*p* < 0.01, ∗∗∗*p* < 0.001, and *p* < 0.05 was considered statistically significant.

## 3 Results and discussion

### 3.1 Synthesis and characterization of DHM-Zn and DZ@YM

**Fig. 2:**
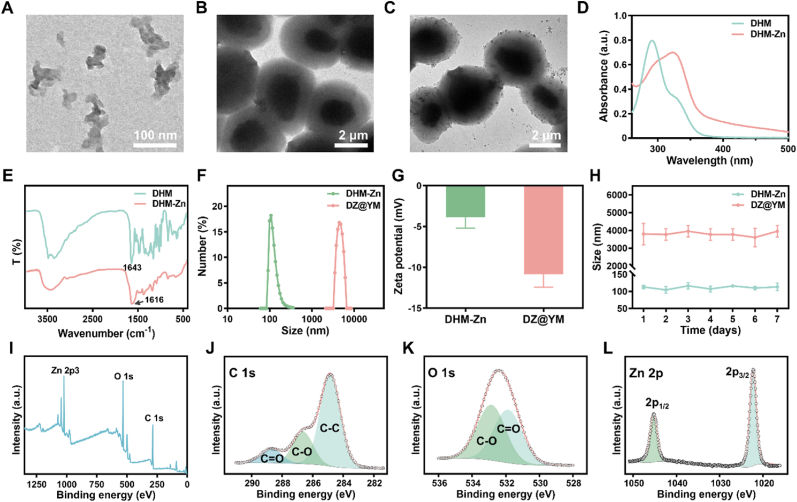
**Characterization of DHM-Zn and DZ@YM.** (A–C) TEM images of DHM-Zn (A), YM (B), and DZ@YM (C). (D) The UV–Vis of DHM and DHM-Zn. (E) FTIR of DHM and DHM-Zn. (F, G) Particle size distribution and zeta-potential of DHM-Zn and DZ@YM. (H) The particle sizes of DHM-Zn and DZ@YM were measured over seven consecutive days. (n = 3). (I–L) XPS of DHM-Zn.

The coordination compound was synthesized through the coordination reaction between DHM and zinc acetate. The microscopic morphology of DHM-Zn was characterized by transmission electron microscopy (TEM), and the results showed that DHM-Zn had a network structure, indicating that the metal-phenolic network was successfully synthesized (Fig. 2A). The TEM results of YM exhibited an elliptical shape with a size of approximately 4 μm (Fig. 2B). The loading of DHM-Zn into YM to prepare DZ@YM resulted in the TEM observations of DHM-Zn on the surface and within the YM structure, thereby confirming the successful synthesis of DZ@YM (Fig. 2C). UV–Vis analysis revealed a red shift in the absorption peak of DHM-Zn from 290 nm to 320 nm, suggesting enhanced conjugation following HO-C complexation in ligand DHM, thereby reducing the transition energy (Fig. 2D). Furthermore, FTIR revealed that the C=O peak of DHM was 1643 cm^−1^, and the C=O peak of DHM-Zn shifted to 1616 cm^−1^, indicating that Zn^2+^ complexed with C=O and formed a coordination compound (Fig. 2E). Concurrently, the particle size and potential of DHM-Zn and DZ@YM were measured by dynamic light scattering. The results showed that the particle size of DHM-Zn was 110.08 ± 5.22 nm, and that of DZ@YM was 4244.92 ± 172.31 nm (Fig. 2F–S1A). The zeta-potentials were −3.88 ± 1.30 mV and −10.83 ± 1.58 mV, respectively (Fig. 2G). To evaluate the stability of DHM-Zn and DZ@YM, the particle size and zeta potential of them were measured continuously for 7 days. The results show that there are no significant changes in both the particle size and zeta potential (Fig. 2H–S1B). Similarly, the TEM results also indicate that there are no obvious morphological changes in DHM-Zn and DZ@YM after being placed for 7 days (Fig. S1C and D). XPS spectra of DHM-Zn showed peaks of Zn 2p3, O 1s, and C1s at 1022, 532, and 285 eV, respectively (Fig. 2I). In the spectrum of C 1s, the binding energy peaks at 284.8, 286.6, and 288.7 eV can be attributed to C-C, C-O, and C=O, respectively (Fig. 2J). In the spectrum of O 1s, the binding energy peaks at 531.9 and 532.8 eV are C=O and C-O (Fig. 2K). Similarly, in the spectrum of Zn 2p, the binding energy peaks at 1022.2 and 1045.3 eV belong to Zn 2p_3/2_ and Zn 2p_1/2_ (Fig. 2L). Taken together, our thorough and systematic structural characterization confirmed that DHM-Zn and DZ@YM were successfully synthesized.

### 3.2 Enzymatic activities of DHM-Zn

**Fig. 3:**
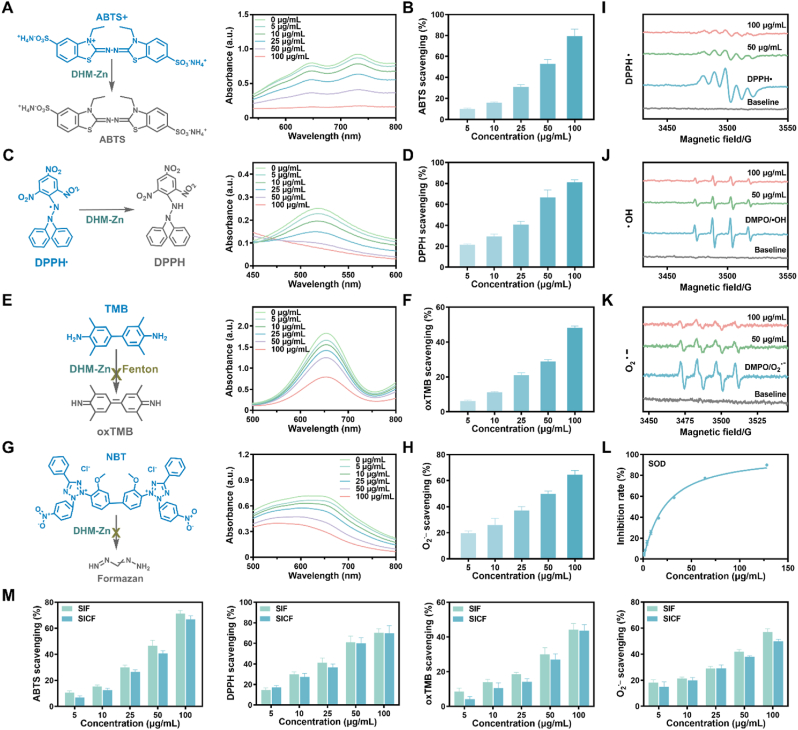
**Enzymatic activities of DHM-Zn.** (A, B) ABTS radical scavenging of DHM-Zn. (C, D) DPPH radical scavenging of DHM-Zn. (E, F) •OH scavenging of DHM-Zn. (G, H) O_2_^•-^ scavenging of DHM-Zn. (I–K) The ability of DHM-Zn to scavenge free radicals was measured by ESR. (L) The SOD assay kit was employed to quantitatively assess the superoxide anion-scavenging capacity of DHM-Zn nanozymes. (M) Free radical scavenging ability of DHM-Zn after incubation with SIF and SICF for 2 h (n = 3).

To verify the antioxidant capacity of DHM-Zn, its enzymatic activity was evaluated. 2,2′-azino-bis (3-ethylbenzothiazoline-6-sulfonic acid) (ABTS) and 1,1-diphenyl-2-picrylhydrazyl (DPPH) free radical scavenging tests are commonly used and validated methods to assess total antioxidant capacity (Fig. 3A–C). The experimental results demonstrated that the scavenging effect of DHM-Zn on ABTS radical and DPPH radical was concentration-dependent, and the scavenging effect was more than 80 % at the concentration of 100 μg/mL (Fig. 3A–D). 3,3′,5,5′-Tetramethylbenzidine (TMB) serves as a well-established chromogenic probe for •OH detection. In Fenton reaction systems, catalytically generated •OH mediates the oxidative conversion of TMB to its oxidized derivative (oxTMB) through electron transfer processes (Fig. 3E). DHM-Zn has been observed to reduce oxTMB, suggesting a capacity for •OH scavenging (Fig. 3E and F). Nitrotetrazolium blue chloride (NBT), a widely employed O_2_^•-^ detector, reacts with O_2_^•-^ to form formazan (Fig. 3G). The results demonstrated that DHM-Zn reduced formogenase production, thereby reflecting its effective O_2_^•-^ scavenging ability (Fig. 3G and H). Furthermore, electron spin resonance (ESR) spectroscopy revealed that 100 μg/mL of DHM-Zn could significantly reduce the spectral signal intensity of DPPH radicals, •OH, and O_2_^•-^ (Fig. 3I–K). The SOD activity of DHM-Zn was also evaluated using a SOD assay kit, and the results demonstrated that DHM-Zn exhibited considerable O_2_^•-^ scavenging ability (Fig. 3L). To further evaluate the free radical scavenging ability of DHM-Zn nanozymes in the intestinal environment, DHM-Zn was incubated in SIF and SICF for 2 h, respectively. Characterization results revealed negligible alterations in particle size and zeta potential under these conditions, demonstrating the structural integrity of DHM-Zn. Moreover, free radical scavenging assays confirmed that DHM-Zn retained potent antioxidant capacity, highlighting its stability and functionality in intestinal-relevant environments (Fig. 3M–S2).

### 3.3 The intracellular antioxidant and anti-inflammatory effects of DHM-Zn

**Fig. 4:**
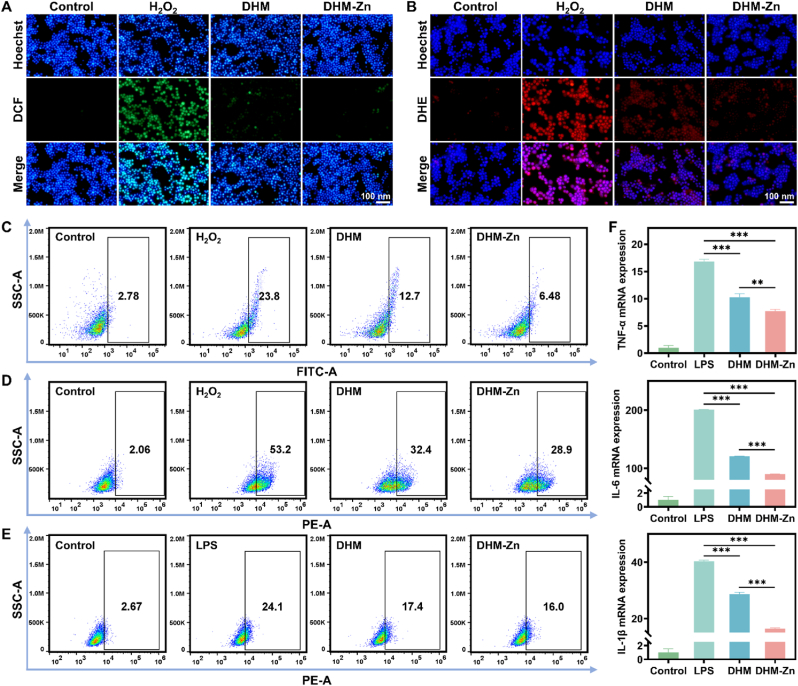
**The intracellular antioxidant and anti-inflammatory effects of DHM-Zn.** (A, B) Fluorescence images of DCF (A) and DHE (B) stained RAW 264.7 cells after different treatments. (C) Flow cytometry analysis of DCF. (D) Flow cytometry analysis of DHE. (E) Flow cytometry analysis of CD86. (F) The mRNA expression of TNF-α, IL-6, and IL-1β in different groups. (n = 3, the significant differences between groups were expressed as ∗*p* < 0.05, ∗∗*p* < 0.01, ∗∗∗*p* < 0.001).

In the context of oxidative stress, a significant amount of ROS is generated within the cell. 2,7-Dichlorofluorescein diacetate (DCFH-DA) is a fluorescent probe employed for the detection of intracellular ROS, which can be converted to DCF with fluorescent characteristics under the oxidation of ROS. Cells exposed to H_2_O_2_ exhibited strong green fluorescence, which intuitively indicated that a significant amount of ROS had been produced in the cells. However, the internal green fluorescence of cells pretreated with DHM and DHM-Zn was significantly weakened, indicating that DHM and DHM-Zn could effectively reduce the level of ROS in the cells (Fig. 4A). Similarly, dihydroethidium (DHE) is a fluorescent probe for O_2_^•-^. Following H_2_O_2_ stimulation, significant red fluorescence was observed in the cells, while DHM and DHM-Zn intervention led to a notable reduction in red fluorescence intensity (Fig. 4B). Subsequent flow cytometry analysis demonstrated that both DHM and DHM-Zn could significantly reduce ROS levels, with the scavenging ability of DHM-Zn being more pronounced (Fig. 4C and D).

Simultaneously, an acute inflammatory model was constructed *in vitro* by induction with LPS. After stimulation with LPS, macrophages could be polarized into M1-type macrophages with pro-inflammatory functions, and these cells were capable of secreting a large amount of pro-inflammatory cytokines. Flow cytometry was used to detect and analyze the specific marker CD86 of M1-type macrophages. The results showed that after induction with LPS, the number of M1-type macrophages increased significantly. In sharp contrast, after treatment with DHM and DHM-Zn, the number of M1-type macrophages decreased significantly (Fig. 4E).

Compared with M1-type macrophages, M2-type macrophages play a crucial role in maintaining the immune homeostasis of the body and promoting the process of tissue repair. To explore the regulatory effects of DHM and DHM-Zn on macrophage polarization under inflammatory conditions, in this study, LPS stimulation was used to simulate the inflammatory environment. By detecting the specific marker CD206 on the surface of M2-type macrophages, an analysis was conducted to determine whether these two substances could promote the transformation of macrophages into M2-type cells to alleviate the inflammatory response. The results of flow cytometry showed that the number of M2-type macrophages decreased significantly after LPS induction. However, after intervention with DHM and DHM-Zn, the decrease in the number of M2-type macrophages caused by LPS induction was significantly reversed (Fig. S3). These results fully indicate that DHM and DHM-Zn can not only significantly inhibit the polarization of macrophages into pro-inflammatory M1-type cells but also effectively promote their transformation into anti-inflammatory M2-type cells, thus playing an important role in reducing the inflammatory response. Furthermore, qPCR analysis quantified transcriptional levels of key pro-inflammatory mediators (TNF-α, IL-6, and IL-1β). When the cells were exposed to LPS, cellular expression profiles of these cytokines demonstrated significant upregulation. However, the DHM and DHM-Zn groups exhibited a marked decrease in cytokine expression compared to the LPS group, with the DHM-Zn group demonstrating a more pronounced reduction (Fig. 4F). These results indicate that DHM-Zn can effectively reduce the expression of inflammatory mediators, thereby alleviating the cellular inflammatory response.

### 3.4 Biosafety assessment of DHM-Zn and DZ@YM

**Fig. 5:**
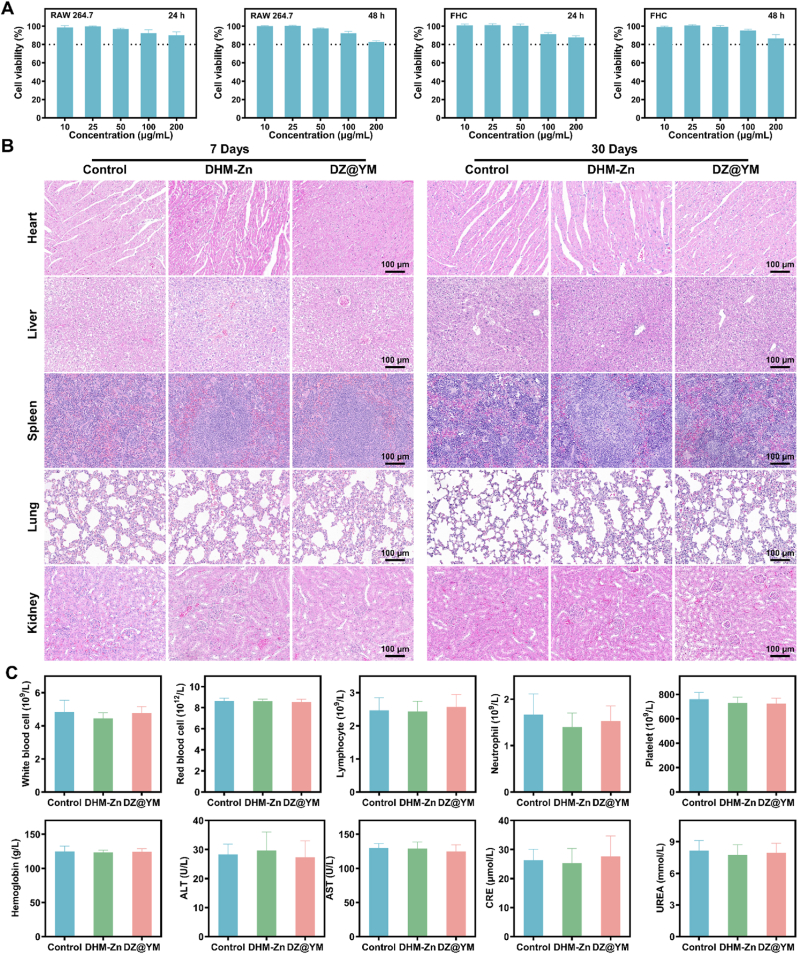
**Biosafety assessment of DHM-Zn and DZ@YM.** (A) Relative viability of RAW 264.7 and FHC cells treated with DHM-Zn for 24 h and 48 h. (B) H&E staining of major organs (heart, liver, spleen, lung, and kidney) isolated from mice receiving oral administration of DHM-Zn and DZ@YM for 7 days and 30 days. (C) Routine blood tests and blood biochemistry were performed in mice following oral administration of DHM-Zn and DZ@YM for 7 days. (n = 3).

Before applying DHM-Zn and DZ@YM to animal models, the biosafety of both was systematically evaluated. Firstly, the MTT method was used to determine the effects of DHM-Zn and DZ@YM on cell viability. The results showed that the viability of RAW 264.7 cells and FHC cells remained stably around 80 % after co-incubation with 200 μg/mL of DHM-Zn for 24 h and 48 h, respectively (Fig. 5A). Similarly, after RAW 264.7 cells were co-incubated with DZ@YM at the same concentration for 24 h and 48 h, the cell viability remained above 80 % (Fig. S4A), which preliminarily confirmed that DHM-Zn and DZ@YM have good cellular safety.

To further explore the acute and chronic toxicity of DHM-Zn and DZ@YM, systematic biosafety tests were conducted 7 days and 30 days after oral administration to mice. Pathological staining and observation of mouse tissues showed that no obvious pathological changes were found either in the short term (7 days) or in the long term (30 days) of administration (Fig. 5B). In terms of hematological index detection, a quantitative analysis of routine blood parameters of mice, such as red blood cell count, white blood cell count, lymphocyte count, platelet count, and hemoglobin concentration, indicated that there was no statistical difference among the DHM-Zn group, the DZ@YM group, and the control group (Fig. 5C–S4B). Meanwhile, liver function indices such as alanine aminotransferase (ALT) and aspartate aminotransferase (AST), as well as renal function indices such as creatinine (CRE) and urea, were also measured. The data showed that there were no significant differences in various indices among the DHM-Zn group, the DZ@YM group, and the control group (Fig. 5C–S4B). The above results fully demonstrate that under the conditions of this study, no acute or chronic toxicity of DHM-Zn and DZ@YM was detected, and both exhibit good biosafety.

### 3.5 The intestinal targeting and retention effects of DZ@YM

**Fig. 6:**
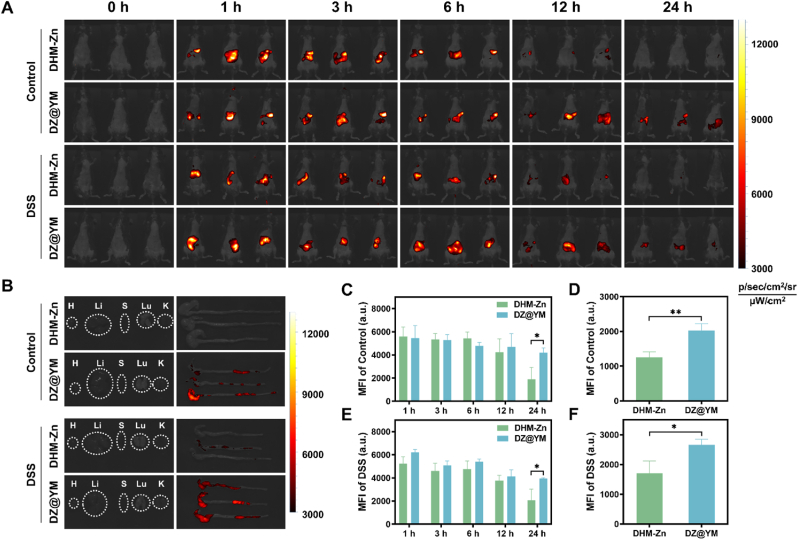
**The intestinal targeting and retention effects of DZ@YM.** (A) Fluorescence images of normal mice and DSS-induced colitis mice at the indicated time points after oral DHM-Zn and DZ@YM. (B) Fluorescence images of colon tissues and organs. H: heart; Li: liver; S: spleen; Lu: lung; K: kidney. (C, D) Quantitative analysis of fluorescence intensity biodistribution in systemic (C) and colonic tissues (D) of healthy mice. (E, F) Quantitative analysis of fluorescence intensity biodistribution in systemic (E) and colonic tissues (F) of DSS-induced colitis mice. (n = 3, the significant differences between groups were expressed as ∗*p* < 0.05, ∗∗*p* < 0.01, ∗∗∗*p* < 0.001).

The gastrointestinal tract presents a dynamic physiological microenvironment for oral drug delivery, where catabolism in the gastric compartment, coupled with active small intestinal absorption, results in subtherapeutic concentrations at colonic target sites. The peristaltic function of the intestine itself leads to a significant reduction in drug residence time. Consequently, these elements may underpin the suboptimal therapeutic outcomes observed in UC. YM is composed of β-glucans, which are capable of withstanding the harsh gastrointestinal environment and delivering drugs to the gut. Furthermore, the *in vivo* retention time of drugs can be effectively prolonged by YM through its specific binding to the dectin-1 receptor on intestinal macrophages [[27], [28], [29]]. To validate the colon-targeting retention capacity of DZ@YM, biodistribution studies were conducted in healthy and DSS-induced colitis murine models through oral gavage administration of DiR-labeled DHM-Zn and DZ@YM. Real-time non-invasive near-infrared imaging revealed distinct pharmacokinetic profiles: In healthy cohorts, the DHM-Zn formulation exhibited complete systemic clearance within 24 h post-administration, whereas DZ@YM demonstrated sustained 24-h luminal retention evidenced by persistent near-infrared signal intensity (Fig. 6A–C). DSS-induced colitis models revealed analogous pharmacokinetic patterns. Despite the gastrointestinal motility dysfunction induced by the disease state, a small amount of DiR-labeled DHM-Zn was still not eliminated from the body at 24 h, while DZ@YM demonstrated a strong retention capacity within the body (Fig. 6A–E). Furthermore, the results of the detection of isolated tissues 24 h after drug administration demonstrated that the DZ@YM group exhibited higher fluorescence intensity in the cecum and colon region, signifying that the number of DZ@YM retained in the cecum and colon was substantial, with minimal distribution observed in other tissues and organs (Fig. 6B). In comparison with the DHM-Zn group, the retention ability of the DZ@YM group in the colon was significantly enhanced, and it showed similar advantages in both normal physiological and inflammatory states (Fig. 6D–F). Similarly, after administering DZ@YM to both healthy mice and mice with colitis, analysis of their colonic tissues revealed significantly higher fluorescence intensity of DZ@YM in the colons of diseased mice compared to healthy controls (Fig. S5). This phenomenon directly indicates that DZ@YM exhibits enhanced retention properties in intestinal tissues under inflammatory conditions, showcasing superior tissue retention capacity compared to the healthy state. These findings strongly confirm that DZ@YM not only demonstrates favorable retention in intestinal tissues but can further augment this effect within the inflamed microenvironment.

### 3.6 Therapeutic efficacy of DZ@YM in DSS-induced colitis

**Fig. 7:**
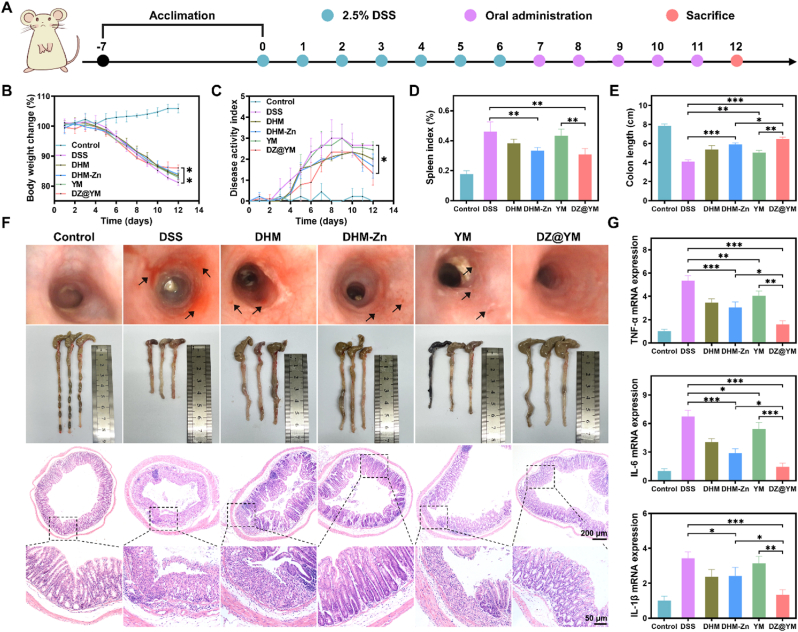
**Therapeutic efficacy of DZ@YM in DSS-induced colitis.** (A) Schematic diagram of the establishment of a DSS-induced colitis mouse model and the arrangement of drug administration time points. (B) The curves of body weight changes of different groups over 12 consecutive days. (C) The curves of DAI scores of different groups over 12 consecutive days. (D) Statistics of the spleen index for different groups. (E) Statistics of the length of the colon for different groups. (F) Endoscopic images, colon images of mice, and images of H&E staining of mice in different groups. (G) The mRNA expression level of pro-inflammatory cytokines (TNF-α, IL-6, IL-1β). (n = 3, the significant differences between groups were expressed as ∗*p* < 0.05, ∗∗*p* < 0.01, ∗∗∗*p* < 0.001).

In the subsequent investigation, the therapeutic efficacy of DZ@YM was assessed in a DSS-induced colitis murine model. The colitis model was established through the continuous administration of DSS to the mice for a period of seven consecutive days. Upon the successful establishment of the model, the mice were then subjected to treatment with the DZ@YM for a duration of five days (Fig. 7A). The daily body weight and disease activity index (DAI) of the mice in the control group demonstrated progressive weight gain with maintained minimal DAI scores, confirming systemic homeostasis and the absence of pathological progression. Conversely, DSS-induced mice exhibited progressive weight loss accompanied by sustained DAI escalation. Therapeutic interventions (four experimental groups) showed attenuated weight reduction and modulated DAI elevation relative to the DSS group. Notably, the DZ@YM group demonstrated a particularly significant response. Following treatment, the body weight of the mice in this group ceased to decrease, while the DAI also underwent a substantial reduction, suggesting that the disease progression in this group was effectively mitigated (Fig. 7B and C). Subsequent endoscopic inspection of the mice demonstrated that the mice within the DSS group manifested diffuse ulcerations in the intestinal mucosal layer, which were accompanied by hemorrhage. Moreover, following treatment with DZ@YM, there was a notable amelioration in the condition of the intestinal tract. This therapeutic effect of DZ@YM was found to be more pronounced than that of DHM-Zn and YM (Fig. 7F). The evaluation of the spleen index demonstrated that DHM-Zn and DZ@YM could significantly reduce the spleen index (Fig. 7D). In addition, both DHM-Zn and DZ@YM demonstrated the capacity to effectively restore colon length. However, DZ@YM exhibited superior efficacy in this regard, while YM exhibited a certain therapeutic effect. However, the efficacy of these treatments was found to be limited (Fig. 7E and F). Further observation of the pathological sections of the colon revealed that the epithelial cells and crypts in the DSS group were all destroyed, accompanied by a large number of inflammatory cell infiltrations. In contrast, the YM group and the DHM-Zn group exhibited preservation of crypt and epithelial cell structure, accompanied by a reduction in the area of lesion (Fig. 7G). The DZ@YM group demonstrated a significant reduction in the degree of colonic pathological damage (Fig. 7F). Moreover, the quantification of pro-inflammatory cytokines through qPCR revealed that the mice in the DSS group displayed significantly increased concentrations of TNF-α, IL-6, and IL-1β. In contrast, both the YM group and the DHM-Zn group showed the ability to reduce the expression levels of these pro-inflammatory cytokines, indicating a potential anti-inflammatory effect. Notably, the DZ@YM group exhibited an enhanced anti-inflammatory effect, characterized by a substantial reduction in the levels of inflammatory factors and a significant alleviation of the development of UC (Fig. 7G).

### 3.7 Modulating gut microbiota by DZ@YM

**Fig. 8:**
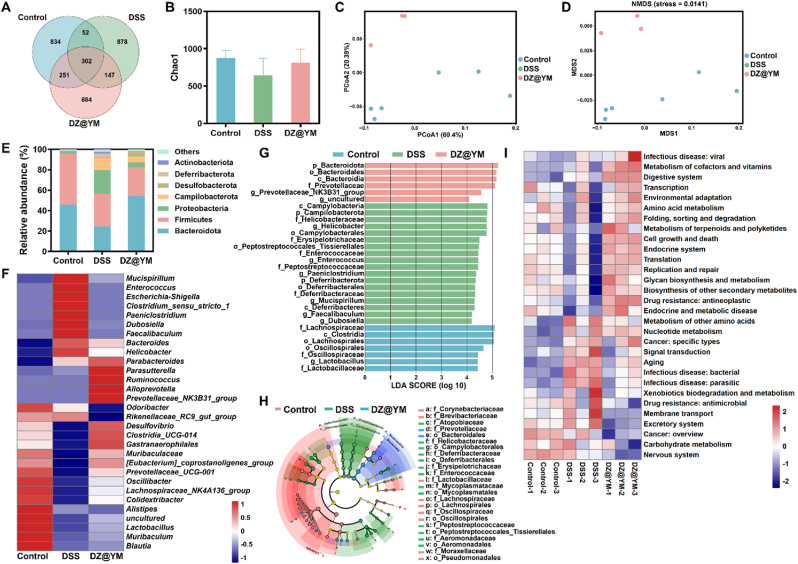
**Modulating gut microbiota by DZ@YM.** (A) The Venn diagrams. (B) The species abundance of gut microbiota is illustrated by Chao1. (C) β-diversity of gut microbiota is demonstrated by PCoA. (D) β-diversity of gut microbiota is demonstrated by NMDS. (E) The abundance at the phylum level. (F) Heatmap of the relative abundance of the genus level. (G) LEfSe analysis. (G) Cladogram based on LEfSe analysis. (I) Heatmap of the KEGG function prediction results. (n = 3).

Gut microbiota plays a pivotal role in the pathogenesis of UC, exerting a profound influence on the disease process by affecting the host's energy metabolism and regulating immune homeostasis. Dysregulation of gut microbiota has been demonstrated to promote the development of UC. The stability of gut microbiota and the loss of biodiversity in UC patients are decreased, which may be related to the maintenance of the integrity of the intestinal biological barrier and the decrease of the regulatory function of the host immune system [30,31]. The intestinal epithelial mucosa is populated by dense microbiota, and the mucus layer and intestinal epithelial cells collaborate to form a physical barrier that maintains the symbiotic relationship between the intestinal microbiota and the host [32]. However, defects in epithelial cells are often observed in patients with inflammatory bowel disease. The disruption of the intestinal barrier has been shown to result in a shift in the composition and structure of the intestinal bacterial community, which in turn can lead to dysfunctional gut microbiota and subsequent exacerbation of intestinal inflammation [33,34]. The analysis of changes in gut microbiota using 16S rRNA gene sequencing revealed a change in ASVs composition following DSS induction (Fig. 8A). Species diversity analysis showed that species abundance and α diversity were reduced in the DSS group (Fig. 8B–S6). Concomitantly, β diversity analysis employing principal coordinate analysis (PCoA) and non-metric multi-dimensional scaling (NMDS) techniques revealed disparities in species composition between the groups (Fig. 8C and D). This finding suggests that DSS-induced changes in the structural and functional properties of gut microbiota. Taxonomic stratification at phylum and genus resolution demonstrated significant β-diversity divergence between the DSS-induced dysbiosis cohort and the DZ@YM therapeutic intervention group (Fig. 8E and F). The abundance of bacteroidota has been demonstrated to be associated with energy metabolism [35]. The results of the study demonstrated a decrease in the abundance of bacteroidota following DSS induction, while the intervention of DZ@YM resulted in an increase in bacteroidota abundance in the gut microbiota. At the genus level, the abundance of beneficial bacteria *parabacteroides* and *muribaculaceae* decreased in the DSS group and increased after DZ@YM intervention. *Parabacteroides*, a constituent of the gut microbiota, has been demonstrated to regulate the immune system, inflammation, metabolism, and lipid metabolism, thereby contributing to the reduction of inflammation [[36], [37], [38]]. *Muribaculaceae* have been shown to produce SCFAs from both endogenous and exogenous polysaccharides, which have been well-documented to exert a crucial function in the attenuation of inflammatory responses within the host biological system [39]. In contrast, the abundance of *clostridium-sensu stricto* and *escherichia Shigella* with pathogenic tendencies increased after DSS induction but decreased after DZ@YM treatment [[40], [41], [42]]. Phylogenetic composition analysis was conducted using LEfSe, and the effect magnitude estimation of differentially abundant species was performed through linear discriminant analysis (LDA) score computation (LDA>4). Among them, bacteroidota and bacteroidales have a significant influence on the differential effect of the DZ@YM group (Fig. 8G and H). Concurrently, the KEGG function prediction identified that DZ@YM exerted a pivotal role in amino acid metabolism, cell growth and death, and replication and repair (Fig. 8I). Consequently, the modulation of the gut microbiota mediated by DZ@YM exerted a synergistic influence on the alleviation of colitis symptoms. This modulation led to an increase in the bacterial diversity within the gut microbiota and induced a compositional shift of the microbiota towards an anti-inflammatory phenotype.

Emerging evidence underscores the pivotal role of gut microbiota-derived metabolites in modulating immune responses, particularly through the regulation of macrophage functions. DZ@YM exhibits remarkable efficacy in modulating the composition and structure of the gut microbiota [43]. DZ@YM contributed to the restoration of intestinal microbial balance by increasing the abundance of beneficial bacteria such as *parabacteroides* and *muribaculaceae*, while regulating potentially harmful bacteria like *clostridium-sensu stricto* and *escherichia Shigella*, thereby creating a favorable gut microenvironment conducive to immune homeostasis.

Under normal circumstances, the polarization of macrophages is in a dynamic equilibrium state to maintain the immune homeostasis of the body. When the body is infected by pathogens or suffers from tissue damage, macrophages polarize towards the M1 type to eliminate pathogens and damaged tissues. However, if the activation of M1 macrophages is excessive or lasts for too long, it will lead to the loss of control of the inflammatory response. In contrast, the timely activation of M2 macrophages helps to control inflammation, promote tissue repair, and restore the body to its normal state. Therefore, regulating the polarization direction of macrophages is an important strategy for treating inflammatory diseases [44]. Notably, the study reveals that DHM-Zn not only inhibits the polarization of macrophages towards the pro-inflammatory M1 phenotype but also actively promotes their transition towards the anti-inflammatory M2 state, underscoring its multifaceted role in inflammation resolution and immune regulation. Consequently, DZ@YM can not only regulate the composition and function of the gut microbiota and reshape the balance of the gut microecology but also regulate the polarization of macrophages and promote their transformation into an anti-inflammatory phenotype. This strategy integrates the dual mechanisms of microecological reshaping and immune regulation, providing a novel therapeutic strategy for inflammatory diseases.

## 4 Conclusion

In conclusion, this study effectively incorporated DHM-Zn nanozymes into YM, resulting in the synthesis of DZ@YM, which exhibits distinct properties. The intestinal targeting capability of YM facilitates the delivery of DHM-Zn to sites of inflammation, thereby maximizing its anti-inflammatory and antioxidant effects. Simultaneously, DZ@YM can modulate the composition and structure of the gut microbiota, synergistically enhancing the intestinal microecological environment and significantly alleviating the symptoms of UC. This approach establishes a novel framework for the future development of microbial-derived drug delivery systems aimed at addressing other intestinal inflammatory disorders.

## CRediT authorship contribution statement

**Meihong Chai:** Writing – original draft, Software, Methodology, Investigation, Formal analysis, Data curation. **Yuanyuan Zhu:** Writing – review & editing, Methodology, Formal analysis, Data curation, Conceptualization. **Liyuan Chen:** Methodology, Formal analysis, Conceptualization. **Shanli Zhang:** Methodology, Investigation, Data curation. **Yahui Huang:** Supervision, Resources, Project administration, Funding acquisition. **Mingzhen Zhang:** Writing – review & editing, Supervision, Resources, Project administration, Funding acquisition, Conceptualization. **Weiwei Jin:** Resources, Funding acquisition.

## Funding

This work was supported by the 10.13039/501100001809National Natural Science Foundation of China (No. 82472127), 10.13039/501100017596Natural Science Basic Research Program of Shaanxi (No. 2024JC-YBQN-0929), Xi'an Science and Technology Plan Project (No. 23YXYJ0067), Xi'an Administration of Traditional Chinese Medicine Project (No. SZZ202402) and the “Young Talent Support Plan” of Xi'an Jiaotong University, China (No. YX6J001).

## Declaration of competing interest

The authors declare that they have no known competing financial interests or personal relationships that could have appeared to influence the work reported in this paper.

## Data Availability

Data will be made available on request.

## References

[1] Le Berre C., Honap S., Peyrin-Biroulet L. (2023). Ulcerative colitis. Lancet.

[2] Kobayashi T., Siegmund B., Le Berre C., Wei S.C., Ferrante M., Shen B., Bernstein C.N., Danese S., Peyrin-Biroulet L., Hibi T. (2020). Ulcerative colitis. Nat. Rev. Dis. Primers.

[3] Chang J.T. (2020). Pathophysiology of inflammatory bowel diseases. N. Engl. J. Med..

[4] Caruso R., Lo B.C., Nunez G. (2020). Host-microbiota interactions in inflammatory bowel disease. Nat. Rev. Immunol..

[5] de Lange K.M., Moutsianas L., Lee J.C., Lamb C.A., Luo Y., Kennedy N.A., Jostins L., Rice D.L., Gutierrez-Achury J., Ji S.G., Heap G., Nimmo E.R., Edwards C., Henderson P., Mowat C., Sanderson J., Satsangi J., Simmons A., Wilson D.C., Tremelling M., Hart A., Mathew C.G., Newman W.G., Parkes M., Lees C.W., Uhlig H., Hawkey C., Prescott N.J., Ahmad T., Mansfield J.C., Anderson C.A., Barrett J.C. (2017). Genome-wide association study implicates immune activation of multiple integrin genes in inflammatory bowel disease. Nat. Genet..

[6] Lopes E.W., Chan S.S.M., Song M., Ludvigsson J.F., Hakansson N., Lochhead P., Clark A., Burke K.E., Ananthakrishnan A.N., Cross A.J., Palli D., Bergmann M.M., Richter J.M., Chan A.T., Olen O., Wolk A., Khalili H., investigators E.-I. (2023). Lifestyle factors for the prevention of inflammatory bowel disease. Gut.

[7] Gros B., Kaplan G.G. (2023). Ulcerative colitis in adults: a review. JAMA.

[8] Sun Y., Liu S.S., Yang S.W., Chen C., Yang Y.T., Lin M.Y., Liu C., Wang W.M., Zhou X.D., Ai Q.D., Wang W., Chen N.H. (2022). Mechanism of dihydromyricetin on inflammatory diseases. Front. Pharmacol..

[9] Zhang Q.L., Zhao Y.F., Zhang M.Y., Zhang Y.L., Ji H.F., Shen L. (2021). Recent advances in research on vine tea, a potential and functional herbal tea with dihydromyricetin and myricetin as major bioactive compounds. J. Pharm. Anal..

[10] Dong S.J., Zhu M., Wang K., Zhao X.Y., Hu L.L., Jing W.H., Lu H.T., Wang S.C. (2021). Dihydromyricetin improves DSS-induced colitis in mice via modulation of fecal-bacteria-related bile acid metabolism. Pharmacol. Res..

[11] Liu D., Mao Y.Q., Ding L.J., Zeng X.A. (2019). Dihydromyricetin: a review on identification and quantification methods, biological activities, chemical stability, metabolism and approaches to enhance its bioavailability. Trends Food Sci. Technol..

[12] Geng H., Zhong Q.Z., Li J., Lin Z., Cui J., Caruso F., Hao J. (2022). Metal ion-directed functional metal-phenolic materials. Chem. Rev..

[13] Yuan H.T., Wang F.J., Wang Z., Gu D., Huang W., Fu C.J., Wang X.X., Ma J.B., Li Z.J., Dai L.Y., Zhang X.Z., Xiao W., Wang J.G. (2023). Natural metal polyphenol nanozyme: free radical scavenging and antioxidation for the treatment of acute kidney injury. ACS Mater. Lett..

[14] Huang W., Tian Y., Ma J., Wei P.H., Du C.Z., Zhang X.D., Chen F.X., Lin Y.X., Zhu Y., Kang D.Z. (2024). Neutrophil membrane-based biomimetic metal-polyphenol self-assembled nanozyme for the targeting treatment of early brain injury following subarachnoid hemorrhage. Chem. Eng. J..

[15] McCallum G., Tropini C. (2024). The gut microbiota and its biogeography. Nat. Rev. Microbiol..

[16] de Vos W.M., Tilg H., Van Hul M., Cani P.D. (2022). Gut microbiome and health: mechanistic insights. Gut.

[17] Lavelle A., Sokol H. (2020). Gut microbiota-derived metabolites as key actors in inflammatory bowel disease. Nat. Rev. Gastroenterol. Hepatol..

[18] Lloyd-Price J., Arze C., Ananthakrishnan A.N., Schirmer M., Avila-Pacheco J., Poon T.W., Andrews E., Ajami N.J., Bonham K.S., Brislawn C.J., Casero D., Courtney H., Gonzalez A., Graeber T.G., Hall A.B., Lake K., Landers C.J., Mallick H., Plichta D.R., Prasad M., Rahnavard G., Sauk J., Shungin D., Vázquez-Baeza Y., White R.A., Braun J., Denson L.A., Jansson J.K., Knight R., Kugathasan S., McGovern D.P.B., Petrosino J.F., Stappenbeck T.S., Winter H.S., Clish C.B., Franzosa E.A., Vlamakis H., Xavier R.J., Huttenhower C. (2019). Multi-omics of the gut microbial ecosystem in inflammatory bowel diseases. Nature.

[19] Sorbara M.T., Pamer E.G. (2022). Microbiome-based therapeutics. Nat. Rev. Microbiol..

[20] Bethlehem L., Estevinho M.M., Grinspan A., Magro F., Faith J.J., Colombel J.F. (2024). Microbiota therapeutics for inflammatory bowel disease: the way forward. Lancet Gastroenterol.

[21] Liu B., Garza D.R., Saha P., Zhou X.J., Faust K. (2024). Exploiting gut microbial traits and trade-offs in microbiome-based therapeutics. Nat Rev Bioeng.

[22] Chen Q.Y., Luo R.F., Han X.Q., Zhang J.M., He Y., Qi S.S., Pu X.L., Nie W.B.A., Dong L.L., Xu H.T., Liu F., Lin M.S., Zhong H.Y., Fu C.M., Gao F. (2021). Entrapment of macrophage-target nanoparticles by yeast microparticles for rhein delivery in ulcerative colitis treatment. Biomacromolecules.

[23] Pu X.L., Ye N.J., Lin M.S., Chen Q.Y., Dong L.L., Xu H.T., Luo R.F., Han X.Q., Qi S.S., Nie W.B., He H.Q., Wang Y.L., Dai L.X., Lin D.S., Gao F. (2021). β-1,3-D-Glucan based yeast cell wall system loaded emodin with dual-targeting layers for ulcerative colitis treatment. Carbohydr. Polym..

[24] Panwar D., Shubhashini A., Kapoor M. (2023). Complex alpha and beta mannan foraging by the human gut bacteria. Biotechnol. Adv..

[25] Tiwari U.P., Fleming S.A., Rasheed M.S.A., Jha R., Dilger R.N. (2020). The role of oligosaccharides and polysaccharides of xylan and mannan in gut health of monogastric animals. J. Nutr. Sci..

[26] Yang J.L., Zhang G.Z., Peng M.Y., Tan S.C., Ge S.C., Yang X.Y., Liang Y., Wen Z.Y., Xie L., Zhou T.H., Wu S.X., An J.Y., Wang Y.F., Liu W., Zhang K.X., Zhang Z.Z., Liu J.J., Shi J.J. (2022). Bionic regulators break the ecological niche of pathogenic bacteria for modulating dysregulated microbiome in colitis. Adv. Mater..

[27] Zhang X.J., Xu X.Q., Chen Y.D., Dou Y., Zhou X., Li L.L., Li C.W., An H.J., Tao H., Hu H.Y., Li X.H., Zhang J.X. (2017). Bioinspired yeast microcapsules loaded with self-assembled nanotherapies for targeted treatment of cardiovascular disease. Mater. Today.

[28] Taylor P.R., Tsoni S.V., Willment J.A., Dennehy K.M., Rosas M., Findon H., Haynes K., Steele C., Botto M., Gordon S., Brown G.D. (2007). Dectin-1 is required for β-glucan recognition and control of fungal infection. Nat. Immunol..

[29] Zhou X., Zhang X.J., Han S.L., Dou Y., Liu M.Y., Zhang L., Guo J.W., Shi Q., Gong G.H., Wang R.B., Hu J., Li X.H., Zhang J.X. (2017). Yeast microcapsule-mediated targeted delivery of diverse nanoparticles for imaging and therapy via the oral route. Nano Lett..

[30] Odenwald M.A., Turner J.R. (2017). The intestinal epithelial barrier: a therapeutic target?. Nat. Rev. Gastroenterol. Hepatol..

[31] Mehandru S., Colombel J.F. (2021). The intestinal barrier, an arbitrator turned provocateur in IBD. Nat. Rev. Gastroenterol. Hepatol..

[32] Horowitz A., Chanez-Paredes S.D., Haest X., Turner J.R. (2023). Paracellular permeability and tight junction regulation in gut health and disease. Nat. Rev. Gastroenterol. Hepatol..

[33] Dunleavy K.A., Raffals L.E., Camilleri M. (2023). Intestinal barrier dysfunction in inflammatory bowel disease: underpinning pathogenesis and therapeutics. Dig. Dis. Sci..

[34] Vancamelbeke M., Vanuytsel T., Farré R., Verstockt S., Ferrante M., Van Assche G., Rutgeerts P., Schuit F., Vermeire S., Arijs I., Cleynen I. (2017). Genetic and transcriptomic bases of intestinal epithelial barrier dysfunction in inflammatory bowel disease. Inflamm. Bowel Dis..

[35] Gibiino G., Lopetuso L.R., Scaldaferri F., Rizzatti G., Binda C., Gasbarrini A. (2018). Exploring Bacteroidetes: metabolic key points and immunological tricks of our gut commensals. Dig. Liver Dis..

[36] Wang K., Liao M.F., Zhou N., Bao L., Ma K., Zheng Z.Y., Wang Y.J., Liu C., Wang W.Z., Wang J., Liu S.J., Liu H.W. (2019). Parabacteroides distasonis alleviates obesity and metabolic dysfunctions via production of succinate and secondary bile acids. Cell Rep..

[37] Koh G.Y., Kane A., Lee K., Xu Q.B., Wu X., Roper J., Mason J.B., Crott J.W. (2018). Parabacteroides distasonis attenuates toll-like receptor 4 signaling and Akt activation and blocks colon tumor formation in high-fat diet-fed azoxymethane-treated mice. Int. J. Cancer.

[38] Koh G.Y., Kane A., Wu X., Crott J.W. (2020). Attenuates tumorigenesis, modulates inflammatory markers and promotes intestinal barrier integrity in azoxymethane-treated A/J mice. Carcinogenesis.

[39] Zhu Y.Q., Chen B.R., Zhang X.Y., Akbar M.T., Wu T., Zhang Y.Y., Zhi L., Shen Q. (2024). Exploration of the family in the gut microbiota: diversity, metabolism, and function. Nutrients.

[40] Udaondo Z., Duque E., Ramos J.L. (2017). The pangenome of the genus Clostridium. Environ. Microbiol..

[41] He X.X., Li Y.H., Yan P.G., Meng X.C., Chen C.Y., Li K.M., Li J.N. (2021). Relationship between clinical features and intestinal microbiota in Chinese patients with ulcerative colitis. World J. Gastroenterol..

[42] Zhang Z., Taylor L., Shommu N., Ghosh S., Reimer R., Panaccione R., Kaur S., Hyun J.E., Cai C., Deehan E.C., Hotte N., Madsen K.L., Raman M. (2020). A diversified dietary pattern is associated with a balanced gut microbial composition of faecalibacterium and Escherichia/Shigella in patients with Crohn's disease in remission. J. Crohns Colitis.

[43] Zhang H., Wang X., Zhang J., He Y., Yang X., Nie Y., Sun L. (2023). Crosstalk between gut microbiota and gut resident macrophages in inflammatory bowel disease. J Transl Int Med.

[44] Yan L., Wang J., Cai X., Liou Y.C., Shen H.M., Hao J., Huang C., Luo G., He W. (2020). Macrophage plasticity: signaling pathways, tissue repair, and regeneration. MedComm.

