# Supplementary material for: Yeast microcapsules encapsulating metal-phenolic nanozymes alleviate ulcerative colitis by mitigating oxidative stress and modulating the gut microbiota
Source: Mater Today Bio. 2025 May 23;32:101902. doi: 10.1016/j.mtbio.2025.101902 (PMC12166400; doi:10.1016/j.mtbio.2025.101902)
Supplement: Multimedia component 1 [file mmc1.docx]

**Supporting Information**

**Yeast microcapsules encapsulating metal-phenolic nanozymes ameliorate ulcerative colitis by mitigating oxidative stress and modulating the gut microbiota**

Meihong Chai^1#^, Yuanyuan Zhu^2#^, Liyuan Chen^2^, Shanli Zhang^2^, Yahui Huang^1*^, Mingzhen Zhang^2*^, Weiwei Jin^3*^

Affiliations:

1. Department of Pharmacy, Xi’an Hospital of Traditional Chinese Medicine, Xi’an, Shaanxi, 710021, China
2. School of Basic Medical Sciences, Xi'an Jiaotong University, Xi’an, Shaanxi, 710061, China
3. General Surgery, Cancer Center, Department of Gastrointestinal and Pancreatic Surgery, Zhejiang Provincial People’s Hospital (Affiliated People’s Hospital), Hangzhou Medical College, Hangzhou, Zhejiang, 310014, China

# These authors contributed equally to this article.

* Correspondence authors

Yahui Huang

Email address: hyhxa1963@126.com

Mingzhen Zhang (ORCID: 0000-0002-4686-6526)

Email address: mzhang21@xjtu.edu.cn

Weiwei Jin (ORCID: 0000-0002-2974-9598)

Email address: jinww@zju.edu.cn

**
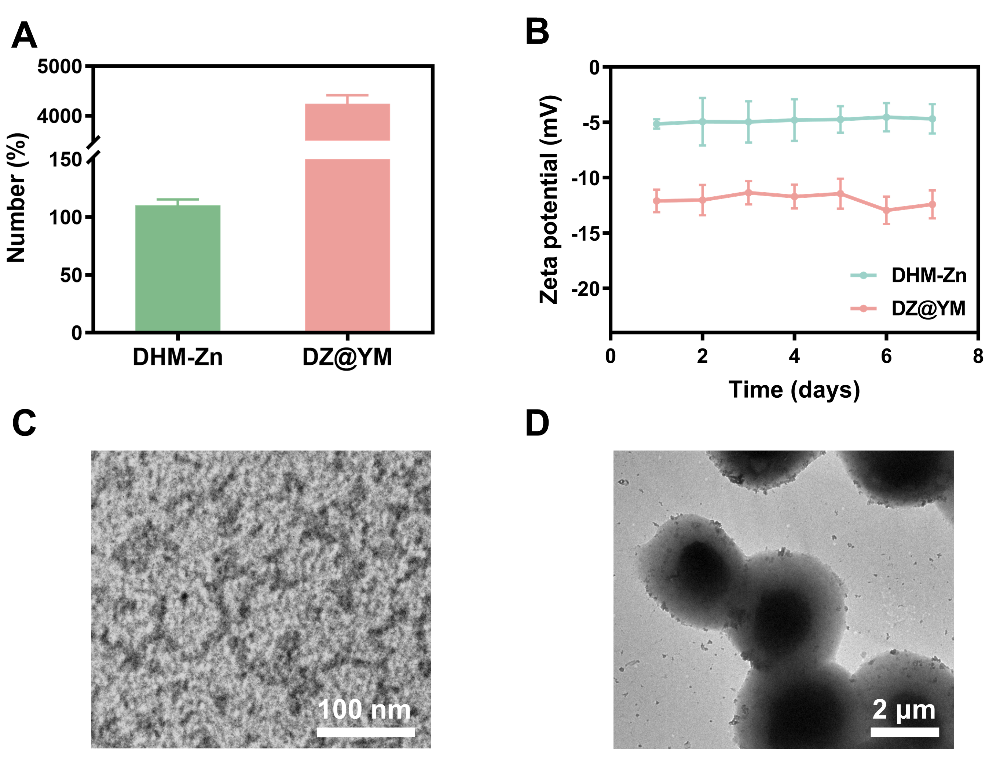
**

**Figure S1. Characterization of DHM-Zn and DZ@YM.** (A) Particle size plot of DHM-Zn and DZ@YM. (n = 3). (B) The zeta-potential of DHM-Zn and DZ@YM was measured over seven consecutive days. (n = 3). (C, D) The TEM images of DHM-Zn (C) and DZ@YM (D).


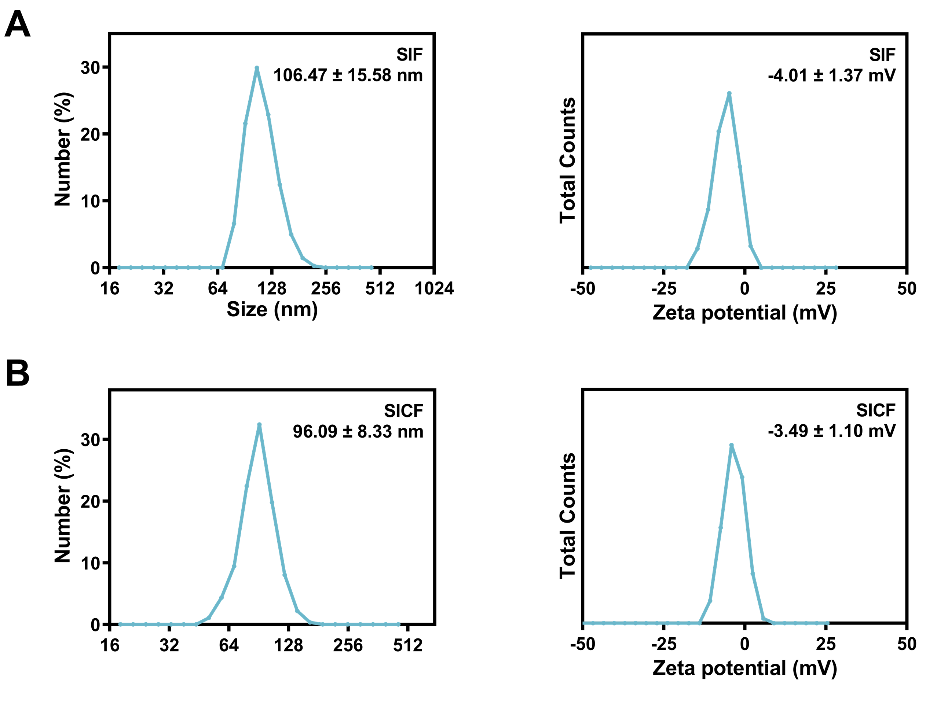


**Figure S2.** **Particle size distribution and zeta-potential of DHM-Zn** **after incubation with SIF and SICF.** (A) Particle size distribution and zeta-potential of DHM-Zn after incubation with SIF for 2 h. (n = 3). (B) Particle size distribution and zeta-potential of DHM-Zn after incubation with SICF for 2 h. (n = 3).


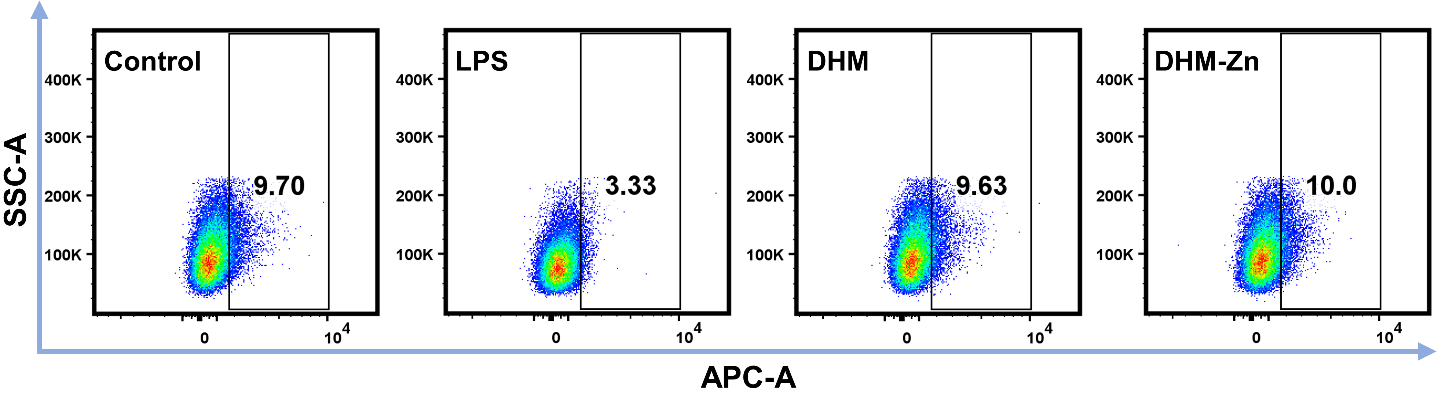


Figure S3. Flow cytometry analysis of CD206.


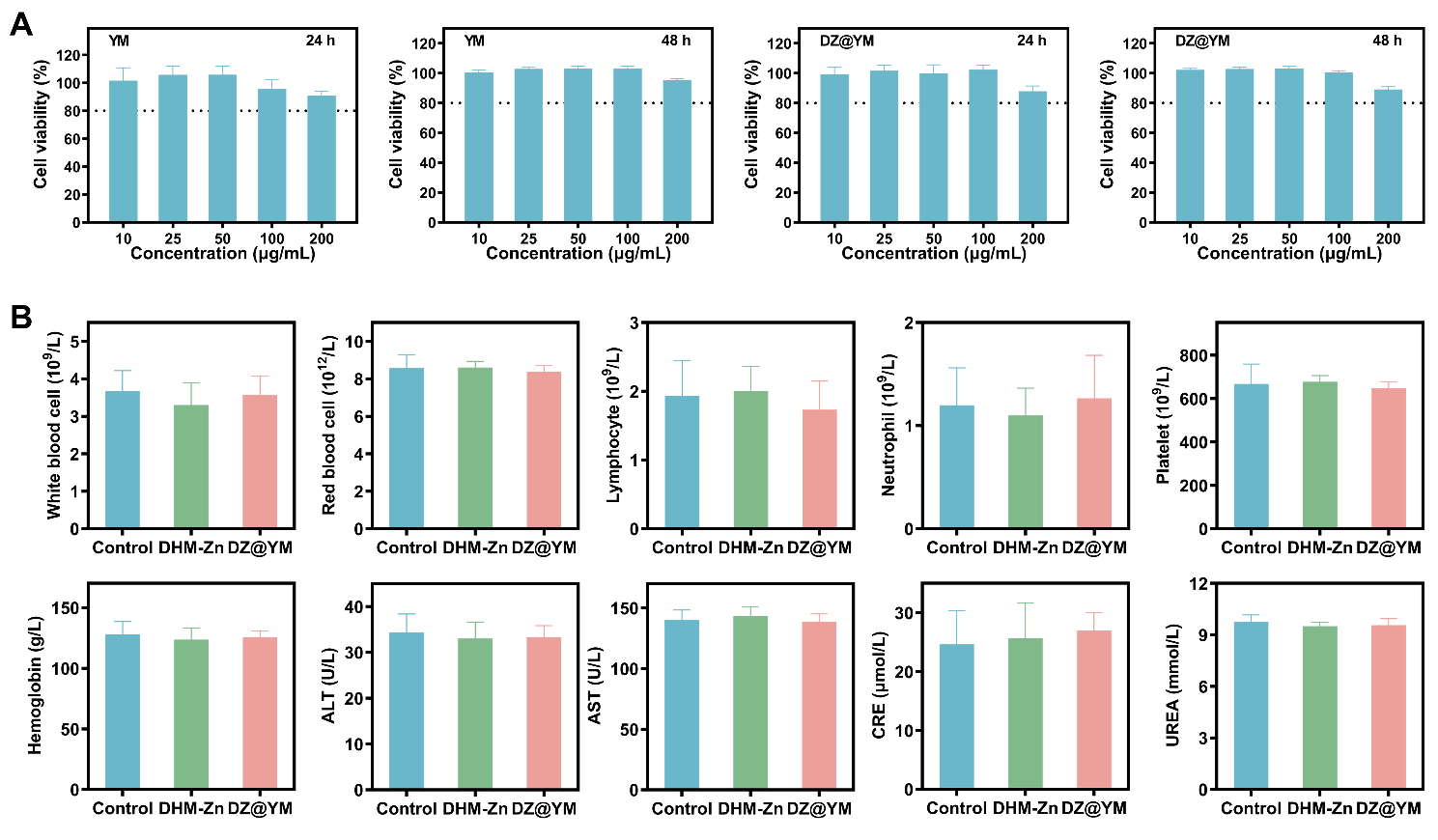


**Figure S4. Biosafety assessment of DHM-Zn, YM, and DZ@YM.** (A) Relative viability of RAW 264.7 cells treated with YM and DZ@YM for 24 h and 48 h. (n = 6). (B) Routine blood tests and blood biochemistry were performed in mice following oral administration of DHM-Zn and DZ@YM for 30 days. (n = 3).


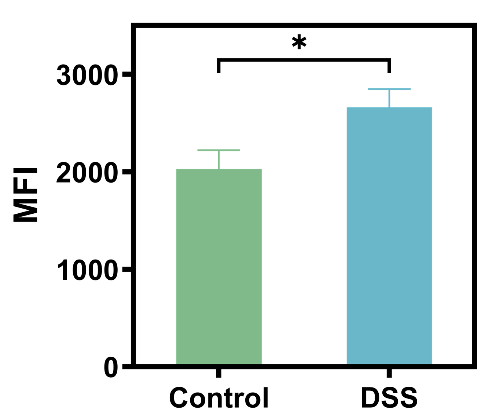


**Figure S5. MFI in the colons of the DZ@YM group**. The mean fluorescence intensity of the DZ@YM group in the colons of normal mice and DSS-induced colitis mice. (n = 3).


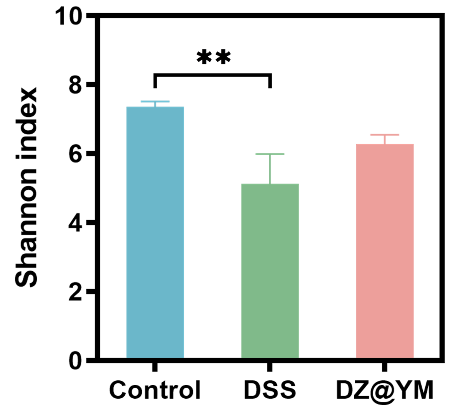


**Figure S6.** **Shannon diversity index** **of gut microbiota.** Analysis of the α-diversity of the gut microbiota by the Shannon diversity index. (n = 3).
